# Mechanism of DNA damage responses induced by exposure to an oligonucleotide homologous to the telomere overhang in melanoma

**DOI:** 10.18632/oncotarget.1047

**Published:** 2013-06-03

**Authors:** Ryan T Pitman, Luke Wojdyla, Neelu Puri

**Affiliations:** ^1^ Department of Biomedical Sciences, University of Illinois College of Medicine, Rockford, Illinois

**Keywords:** T-oligo, Tankyrase, TRF1, telomere, p53, melanoma

## Abstract

T-oligo, an 11-base oligonucleotide homologous to the 3'-telomeric overhang, is a novel, potent therapeutic modality in melanoma and multiple other tumor types. T-oligo is proposed to function in a manner similar to experimental disruption of the telomere overhang and induces DNA damage responses including apoptosis, differentiation and senescence. However, important components involved in T-oligo induced responses are not defined, particularly the role of p53, TRF1 and TRF2 in mediating the T-oligo induced responses. In MU, PM-WK, and MM-MC melanoma cells, exposure to T-oligo upregulates p53 expression and phosphorylation, resulting in cellular differentiation and activation of a caspase-mediated apoptotic cascade. However, siRNA-mediated knockdown of p53 completely blocks T-oligo induced differentiation and significantly decreases apoptosis, suggesting that p53 is an important mediator of T-oligo induced responses. In addition, we characterized the roles of telomere binding proteins, TRF1, TRF2, and tankyrase-1, in T-oligo induced damage responses. We demonstrate that tankyrase-1 activity is required for initiation of T-oligo induced damage responses including p53 phosphorylation and reduction of cellular proliferation. These results highlight TRF1, TRF2, tankyrase-1 and p53 as important elements in T-oligo mediated responses and suggest new avenues for research into T-oligo's mechanism of action.

## INTRODUCTION

Telomeres and telomerase are areas of active research in tumor biology. Telomeres are structures that serve protective roles by allowing cells to distinguish chromosome ends from damaged DNA [1]. Disruption of the telomere results in multiple adverse consequences including potent DNA damage responses, chromosome fusion via non-homologous end joining, and genome instability [1]. Therefore, targeted disruption of the telomere or telomerase is an attractive therapeutic option [2]. Consequently, administration of T-oligo, an 11-base oligonucleotide homologous to the 3′ telomeric overhang, has been proposed as both a cancer therapeutic [3], and a method to study DNA damage responses induced by disruption of the telomere [4, 5].

Telomeres are composed of tandem nucleotide repeats (TTAGGG) at the end of chromosomes [1] that form a protective T-loop at the single-stranded 3′ end. The T-loop caps and protects the telomere end through recruitment of the shelterin complex, composed of telomeric repeat binding factors 1 and 2 (TRF1 and TRF2), POT1, TIN1, TPP1 and RAP1 [6]. TRF1 and TRF2 directly bind the duplex DNA region of T-loops and function as negative regulators of telomere length by preserving loop structure integrity and preventing telomerase access to the telomere [7-9]. Expression of dominant negative TRF2 induces uncapping of the telomere overhang and initiates DNA damage responses, possibly by disruption of the T-loop and exposure of the telomere overhang, a process mediated in part through ATM and its effector protein p53 [5, 10]. Conversely, overexpression of TRF2 in telomerase negative cells leads to an increased rate of telomere shortening and less efficient repair of single-stranded breaks (SSBs) in telomeric DNA [11]. TRF1 functions in a similar manner by binding telomeric DNA and stabilizing the shelterin complex [6]. Both TRF1 and TRF2 are thought to be key mediators of T-loop integrity and their disruption could lead to downstream DNA damage responses.

Tankyrase-1, a telomere-specific poly(ADP-ribosyl) polymerase (PARP), acts as an inhibitor of TRF1 by catalyzing the addition of poly (ADP-ribose) (PAR) groups to TRF1 and preventing it from binding telomeric DNA [12]. Overexpression of tankyrase-1 leads to telomerase binding and telomere elongation in telomerase positive cells [13], presumably as a consequence of TRF1 inhibition [14, 15]. Consistent with this data, inhibition of tankyrase-1 induces telomere shortening in the presence of telomerase, and thus its inhibition has emerged as a prospective cancer therapy [16]. Tankyrase-1 inhibitors, XAV939 and 3-aminobenzamide (3AB), block telomerase from accessing telomeric DNA [13], presumably by preventing PARsylation of TRF1 [12] and cause a downstream DNA damage response [17, 18].

T-oligo has been proposed as a means for studying downstream DNA damage responses caused by exposure of the telomere overhang. T-oligo induces potent DNA damage responses [4, 19] including transient cell cycle arrest, adaptive differentiation, replicative senescence and apoptosis [3, 4, 20, 21]. T-oligo accumulates in the nucleus and initiates downstream signaling through p95/Nbs1, p16, pRb, p53, p73 and p21 [3, 4, 20-22]. However, induction of DNA damage responses by T-oligo does not require damaged telomeric DNA [19], and T-oligo may lead to an increase in mean telomere length [4]. Further, T-oligo has been proposed as a cancer therapeutic because it induces apoptosis and differentiation and specifically targets malignant cells [3, 5, 23].

At present, it is unclear how introduction of T-oligo into the cell results in a downstream DNA damage response. T-oligo may function as a “replacement” signal in place of telomeric DNA obscured by overexpressed telomerase [5]. We propose that T-oligo initiates a DNA damage response through recruitment of telomeric structures away from the telomere with subsequent exposure of the telomere overhang. To investigate the connection between telomere exposure and T-oligo-induced DNA damage responses, we utilized tankyrase-1 inhibitors, XAV939 and 3AB, to prevent tankyrase-1 mediated dissociation of TRF1 from telomeric DNA. This would, in effect, prevent dissociation of the shelterin complex and disruption of the telomere overhang which, under normal circumstances, induces a DNA damage response [6]. We then exposed cells to T-oligo to determine if tankyrase-1-mediated dissociation of TRF1 from the telomere is required for T-oligo to elicit a DNA damage response.

Further, the exact signaling pathways through which DNA damage responses, mediated by T-oligo, proceed are still undefined. p53, a DNA damage response mediator, has been proposed to play an important role in T-oligo induced DNA damage responses as it is upregulated and phosphorylated in response to T-oligo [5]. However the role of p53 in T-oligo-mediated anticancer responses in melanoma has not been demonstrated. Thus, we investigated the downstream effects of T-oligo, including differentiation and the apoptotic caspase cascade, before and after siRNA mediated p53 knockdown, in p53 expressing MU, PM-WK, and MM-MC melanoma cells.

In this study, we improve our understanding of the DNA damage pathways induced by T-oligo through an examination of the role of p53 in apoptosis and differentiation in melanoma cells. Further, we examine the role of the telomere binding proteins, TRF1 and TRF2, and also delineate the requirement of tankyrase-1 activity for T-oligo's downstream effects.

## RESULTS

### Treatment with T-oligo induces p53 expression and phosphorylation in melanoma cells

**Figure 1:**
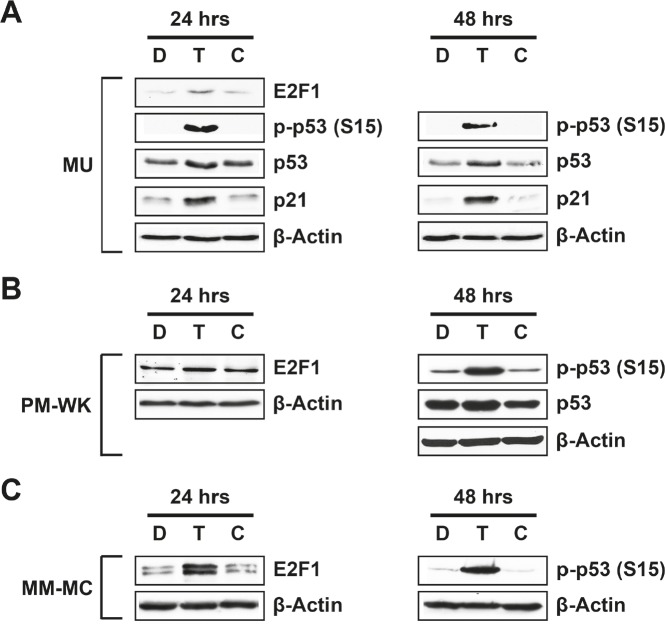
Induction of E2F1, p53, p21 and phosphorylation of p53 (Ser15) in melanoma cells MU, MM-MC or PM-WK melanoma cells were treated with 40 μM of T-oligo (T), complementary oligo (C) or an equal volume of diluent (D) for 24-48 hrs and subsequently prepared for immunoblotting. (A) MU cells demonstrate upregulation of p53 at 24 hrs (1.5-fold) and 48 hrs (2-fold) with corresponding increase in phosphorylation of p53 (ser15) of 18-fold and 25-fold at 24 and 48 hrs, respectively. Further, p21, a downstream transcription product of p53, is increased 2.5-fold and 5-fold in MU cells at 24 and 48hrs, respectively. E2F1 expression is also increased by 2.5-fold at 24 hrs. (B) T-oligo induces a similar increase in p53 and phospho-p53 (ser15) by 1.5-fold and 4.8-fold respectively at 48 hrs in PM-WK cells, with an increase in E2F1 expression by 1.5-fold at 24 hrs in PM-WK cells. (C) MM-MC also exhibit upregulation of E2F1 (3.5-fold) after 24hrs of exposure to T-oligo along with a significant 17-fold increase in p53 phosphorylation after 48 hrs of exposure to T-oligo. Experiments were performed in triplicate with representative results displayed.

Exposure of the telomere overhang induces a strong DNA damage response in malignant cells including apoptosis and differentiation [3-5]. T-oligo induced DNA damage responses are believed to progress through ATM, p53, E2F1 and p95/NBS1 [3, 5, 19, 24], however, the roles of ATM and p53 are unclear [4, 22]. To define the role of p53 in melanoma cells, we exposed p53-expressing MU, MM-MC and PM-WK melanoma cells to T-oligo for various time points and then performed immunoblotting. In MU cells, T-oligo increases E2F1, a driver of p53 expression [25, 26], after 24 hrs of exposure in addition to upregulation of p53 expression and phosphorylation after 24 and 48 hrs of exposure (Fig 1A). T-oligo also upregulates p21, a transcriptional target of p53 [27], after 24 and 48 hrs of exposure in MU melanoma cells (Fig 1A). PM-WK cells exhibit a similar response with increased E2F1 after 24 hrs of exposure to T-oligo and upregulation of p53 expression and its phosphorylation at 48 hrs of exposure (Fig 1B). A similar response is demonstrated in MM-MC cells with significant upregulation of E2F1 after 24 hrs and p53 phosphorylation after 48 hrs of exposure to T-oligo (Fig 1C). These results suggest an integral role of p53 in T-oligo induced DNA damage responses.

### Exposure to T-oligo induces a caspase cascade

**Figure 2:**
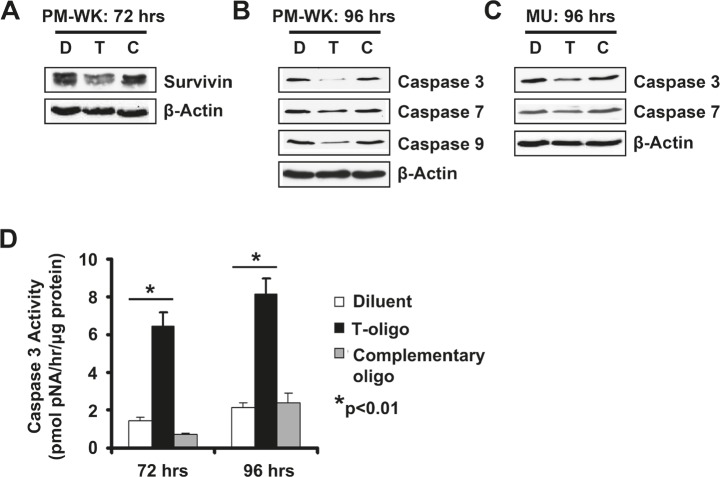
T-oligo initiates a caspase cascade in melanoma cells Melanoma cells were treated with of T-oligo (T), complimentary oligo (C) or an equal volume of diluent (D) for indicated times and cell lysates were immunoblotted for detection of survivin and caspase proenzymes. (A) T-oligo treatment in PM-WK caused downregulation in survivin (1.5-fold) (B) followed by a 9.3, 2.9, and 1.5-fold decrease in caspase-3, 7, and 9 proenzymes respectively, at 96 hrs of incubation with T-oligo as compared to the complimentary oligonucleotide. (C) Similarly, MU melanoma cells also demonstrate a decrease in caspase-3 (3.3-fold) and 7 (2.6-fold) at 96 hrs of treatment with T-oligo as compared to the complementary oligo. (D) To confirm increased caspase-3 catalytic activity in response to T-oligo treatment, MU cells were treated as described above for 72 or 96 hrs and subsequently collected and analyzed for caspase-3 activity using the CaspACE assay system. Caspase-3 activity is increased by 4.5-fold and 4-fold at 72 and 96 hrs, respectively. Immunoblots are representative of at least three independent experiments. Analysis of caspase-3 activity was done in triplicate with error bars representing standard deviation. (* = p<0.01)

Studies in our laboratory and others show that T-oligo selectively induces apoptosis in multiple types of malignant cells including melanoma, prostate, NSCLC, ovarian and breast [3-5, 22, 23, 28-30]. However, few insights are available into the involvement of an apoptotic cascade in T-oligo induced apoptosis in melanoma. To further define and understand the caspases induced by T-oligo in p53-expressing melanoma cells, we analyzed MU and PM-WK melanoma cells for induction of a caspase cascade. In PM-WK cells, survivin, an inhibitor of apoptosis that is overexpressed in melanoma and blocks activation of caspase-9 [31, 32], is downregulated after 72 hrs of exposure to T-oligo (Fig 2A), with subsequent decrease of the caspase-9, caspase-3, and caspase-7 proenzymes after 96 hrs of treatment with T-oligo (Fig 2B). In addition, MU cells also exhibit a decrease in caspase-3 and caspase-7 proenzymes after 96 hrs of exposure to T-oligo (Fig 2C). To validate and quantify caspase-3 activation and activity, we measured caspase-3 catalytic activity using the colorimetric CaspACE assay. In MU cells, increased caspase-3 activity is observed after both 72 and 96 hrs of treatment with T-oligo (Fig 2D). These results establish involvement of the caspase cascade in T-oligo induced apoptosis in melanoma.

### Induction of melanoma differentiation after exposure to T-oligo

Although much attention is given to T-oligo induced apoptosis, the development of metastatic melanoma is often coupled with a loss of melanocyte differentiation-specific antigens [33] that are utilized in immunotherapy-mediated treatment of melanoma [34]. Previous studies identify an important link between exposure of the telomere overhang with differentiation in melanoma [35-37], and we suggest that T-oligo could also mediate differentiation of melanoma cells [3]. Additionally, differentiation of melanoma cells is associated with slowed growth and reduced tumorigenicity [36, 37] and is the target of melanoma vaccine therapy [34].

**Figure 3:**
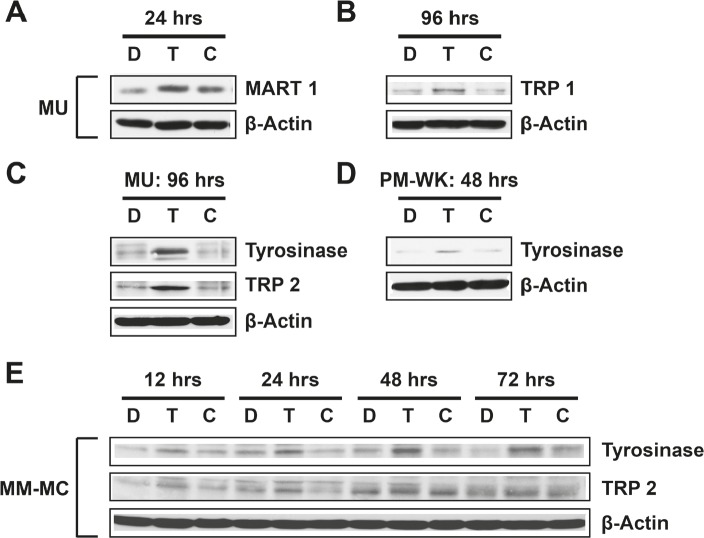
T-oligo strongly induces differentiation in multiple melanoma cell lines Melanoma cells were exposed to 40 μM of T-oligo (T), complementary oligo (C) or an equal volume of diluent (D) for the indicated times and samples were collected for immunoblotting. (A) In MU melanoma cells, T-oligo upregulates MART-1 by 2.7-fold at 24 hrs, (B) and TRP-1 by 2.5-fold at 96 hrs. (C) Additional upregulation of TRP-2 (2.9-fold) and tyrosinase (2.3-fold) is observed in MU cells after 96 hrs of treatment with T-oligo. (D) PM-WK cells also demonstrate 3-fold increase in tyrosinase after only 48 hrs of exposure to T-oligo. (E) To better understand the time course of T-oligo-induced differentiation, MM-MC melanoma cells were immunoblotted for the expression of TRP-1 and TRP-2 over the course of 72 hrs of exposure to T-oligo. Both TRP-1 and TRP-2 are increased over the entire course of 12 to 72 hrs of T-oligo treatment (2.9-fold and 2.6-fold, respectively). Immunoblots are representative of at least three independent experiments.

Currently, it is unclear if treatment with T-oligo can cause uniform differentiation across all melanoma cell types. To determine the extent of T-oligo induced differentiation, MU, PM-WK and MM-MC melanoma cells were exposed to T-oligo and subsequently immunoblotted for expression of the melanoma differentiation proteins MART-1, TRP-1, TRP-2, and tyrosinase. MU melanoma cells exposed to T-oligo express MART-1 after 24 hrs (Fig 3A) with additional expression of TRP-1 (Fig 3B), tyrosinase and TRP-2 after 96 hrs (Fig 3C). Interestingly, PM-WK cells express only tyrosinase after 48 hrs of exposure to T-oligo (Fig 3D), while tyrosinase and TRP-2 are increased in MM-MC cells over the entire course of 72 hrs of exposure to T-oligo (Fig 3E). These results provide compelling evidence for strong and progressive T-oligo-induced differentiation in p53-expressing melanoma.

### Loss of T-oligo induced differentiation and apoptosis after p53 knockdown

**Figure 4:**
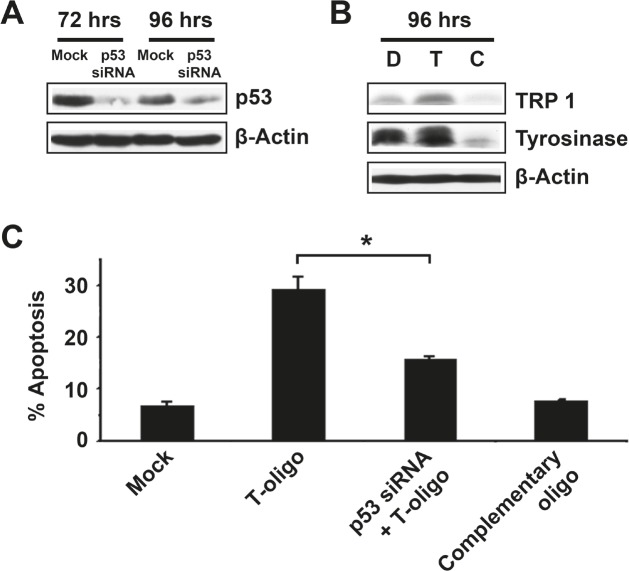
T-oligo induced differentiation and apoptosis in melanoma is dependent upon the presence of p53 MU melanoma cells were transfected with p53 specific siRNA or mock siRNA and analyzed using immunoblotting. (A) Down regulation of p53 expression can be seen at 72 hrs (10-fold) and 96 hrs (2.2-fold). (B) MU cells treated with siRNA against p53 for 12 hrs were then exposed to T-oligo for 96 hrs and immunoblotted for TRP-1 and Tyrosinase. p53 knockdown completely blocks T-oligo-induced differentiation. (C) p53-deficient MU cells were then treated with T-oligo for 72 hrs, collected and stained with propidium iodide for FACS analysis. Knockdown of p53 decreased T-oligo-induced apoptosis by 45%. Immunoblots are representative of at least three independent experiments. Determination of apoptosis was performed in quadruplicate with error bars representing standard deviation. Statistical analysis by the Student's T-test found a significant difference between cells treated with T-oligo and those treated with both siRNA against p53 and T-oligo. (*= p<0.001)

While p53 is proposed to be a primary mediator of T-oligo-induced DNA damage responses in breast cancer [5], the role of p53 in melanoma is unclear as p53-deficient MM-AN cells undergo apoptosis after upregulation of p73 [4]. To demonstrate a definitive role for p53 in melanoma differentiation and apoptosis in response to T-oligo exposure, MU cells were transfected with either anti-p53 siRNA or control siRNA for 12 hrs. Knockdown of p53 expression was confirmed by a 10- and 2.2-fold downregulation at 72 and 96 hrs after transfection, respectively (Fig 4A). MU cells treated with siRNA against cells were then exposed to T-oligo for 72 hrs and subsequently stained with propidium iodide and analyzed by FACS analysis for apoptosis. Loss of p53 decreased T-oligo induced apoptosis by 45% (Fig 4C), indicating that it has a substantial, although perhaps not exclusive, role in apoptosis. Additionally, p53 knockdown eliminated the T-oligo-induced expression of tyrosinase and TRP-1 (Fig 4B). This data suggests that p53 plays a vital role in T-oligo-induced apoptosis and differentiation in melanoma.

### Inhibition of tankyrase-1 blocks T-oligo-induced DNA damage responses

Although the T-oligo induced DNA damage responses are well established [3-5, 19-21, 29], the initiating events that lead to T-oligo induced DNA damage responses are not defined. T-oligo is proposed to function in a manner similar to exposure of the telomere overhang [5] as T-oligo induced DNA damage responses are similar to the effects of TRF2 disruption [5, 19]. TRF1 functions in similar manner as TRF2 by mediating the association of the shelterin complex with the telomere [6] and is PARsylated by tankyrase-1 which leads to its dissociation from the telomere [12], exposure of the telomere overhang and recruitment of telomerase [13]. Inhibitors of tankyrase-1 prevent PARsylation of TRF1 [12] thereby preventing exposure of the telomere overhang with consequences including an inability of telomerase to bind the telomere and subsequent development of senescence [13, 38]. To determine the role of telomere exposure in induction of T-oligo's DNA damage responses, we utilized XAV939 and 3AB, inhibitors of tankyrase-1.

**Figure 5:**
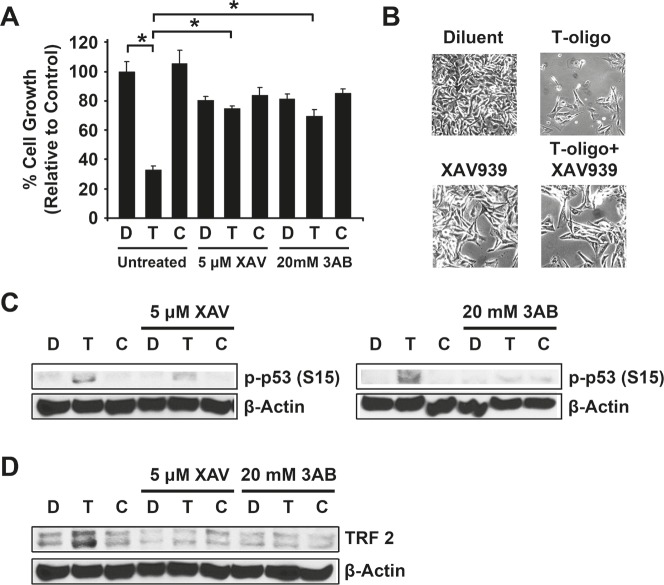
Inhibition of Tankyrase-1 prevents T-oligo induced DNA damage responses MU cells were treated with 5 μM XAV939 or 20 mM 3AB prior to treatment with 40 μM T-oligo or complementary oligo. (A) Treatment with both XAV939 and 3AB decreased cellular proliferation in MU cells. However, treatment of MU cells with XAV939 or 3AB with T-oligo completely blocks T-oligo-induced decrease in cellular proliferation. (B) Microphotographs depicting effect of XAV939 treatment on T-oligo-induced reduction in cellular proliferation. (C) Treatment with XAV939 or 3AB also blocks T-oligo-induced phosphorylation of p53 (ser15). (D) Exposure of MU cells to T-oligo induces a 2.6-fold increase in TRF2 levels, but this effect is also blocked by treatment with XAV939 or 3AB. The effects of T-oligo after XAV939 or 3AB treatment are not significant by analysis using one-way ANOVA. Immunoblots are representative of at least three independent experiments. (*= p<0.001)

MU melanoma cells cultured as described above and treated with XAV939 or 3-aminobenzamide (3AB) demonstrate reduced cellular proliferation (Fig 5A,B) [18]. However, simultaneous treatment with XAV939 or 3AB completely blocked T-oligo-induced inhibition of cellular proliferation since cells treated with T-oligo and 3AB or XAV939 are not significantly different from cells treated with diluent or complementary oligonucleotide and 3AB or XAV939 as determined by one-way ANOVA. Further, treatment with XAV939 or 3AB prevented T-oligo induced p53 phosphorylation (Fig 5C). These results suggest that tankyrase-1 must be able to PARsylate and remove TRF1 from the telomere in order for T-oligo to induce a DNA damage response. Immunoblotting of MU cells treated with T-oligo demonstrate upregulation of TRF2 (Fig 5D), and treatment with XAV939 or 3AB blocked this effect. This is the first demonstration of T-oligo mediated upregulation of TRF2. These results are novel and suggest an important requirement of tankyrase-1 activity leading to TRF1 dissociation from the telomere for induction of T-oligo-mediated DNA damage responses.

## DISCUSSION

Investigation into telomere biology is an important area for understanding tumor biology and identification of novel therapeutic modalities [2]. T-oligo has important therapeutic potential in multiple tumor types, but its mechanism is not fully defined. In this manuscript, we demonstrate induction of a caspase cascade and upregulation of differentiation markers in p53-expressing MU, PM-WK, and MM-MC cells. Further, we verify the importance of p53 in the downstream DNA damage responses induced by T-oligo. We also inhibit tankyrase-1 activity and demonstrate a requirement for tankyrase-1 activity in T-oligo induced DNA damage responses.

p53 expression and phosphorylation are hallmarks of the DNA damage response seen in cells exposed to DNA alkylating agents [39], exposure of the telomere overhang through disruption of the T-loop structure [40], and exposure to T-oligo [4, 20]. However, its role in T-oligo induced apoptosis has been questioned as lack of p53 does not block T-oligo induced apoptosis in cells that do not express p53 [3, 4, 19, 22]. In cells that do express p53, we suggest that p53 is still likely to play an important role in driving DNA damage responses. In this study, p53-expressing melanoma cells undergo a caspase cascade and exhibit signs of differentiation upon exposure to T-oligo. Further, knockdown of p53 completely blocks T-oligo induced differentiation and significantly decreases apoptosis after exposure to T-oligo. However, the inability for p53 knockdown to completely block T-oligo induced apoptosis suggests other pathways likely contribute. p73, a p53 homologue, is upregulated in p53-deficient melanoma cells after exposure to T-oligo and transfection of a dominant-negative p73 significantly decreases T-oligo induced apoptosis [4]. Alternatively, the WRN helicase, which responds to disruption in the telomere overhang, is also suggested to play a role in T-oligo induced apoptosis [24], although the mechanism whereby WRN signals for a DNA damage response is unclear. Although these results suggest that p53 is likely to play an important role in T-oligo induced DNA damage responses, further research is required to understand other key proteins involved in T-oligo induced DNA damage responses.

Despite an increasing understanding of T-oligo's DNA damage responses [4, 19-21] and demonstration of T-oligo's potential as a therapeutic in multiple tumor types [3, 5, 22, 28, 29], little is understood of how T-oligo activates the DNA damage responses. ATM and WRN, a helicase protein, have been implicated [19, 24] as primary drivers, but it is unclear how T-oligo is able to signal for DNA damage. Others have suggested that T-oligo mimics an exposed 3′ telomere overhang, thus initiating the DNA damage pathway [19, 41]. This conclusion is based on observations that the DNA damage responses induced by T-oligo and disruption of the telomere overhang by transfection with a dominant-negative TRF2 follow similar pathways and time-courses [5, 19]. However, administration of T-oligo does not cause degradation of the telomere overhang, seen in TRF2 disruption [19]. Further, these observations do not account for an increase in mean telomere length [4] or transient telomerase activation in response to T-oligo administration [42]. In light of these observations, we suggest that T-oligo does not simply “mimic” the telomere overhang.

TRF1 and TRF2 function to stabilize the telomere overhang through their association with the shelterin complex [6] and tankyrase-1 is proposed to allow telomerase to function through destabilization of shelterin through PARsylation of TRF1 [13, 14]. Tankyrase-1 inhibitors prevent TRF1 dissociation from the telomere [43] and are thought to contribute to apoptosis through increased sister chromatid exchanges at the telomeres [17], or inhibition of telomerase [43]. In this study, we confirm a decrease in cellular proliferation by tankyrase-1 inhibition, but also demonstrate that inhibition of tankyrase-1 abrogates T-oligo-induced p53 phosphorylation and decreased cellular proliferation. These results suggest that tankyrase-1 function is vital to T-oligo mediated anticancer effects.

**Figure 6:**
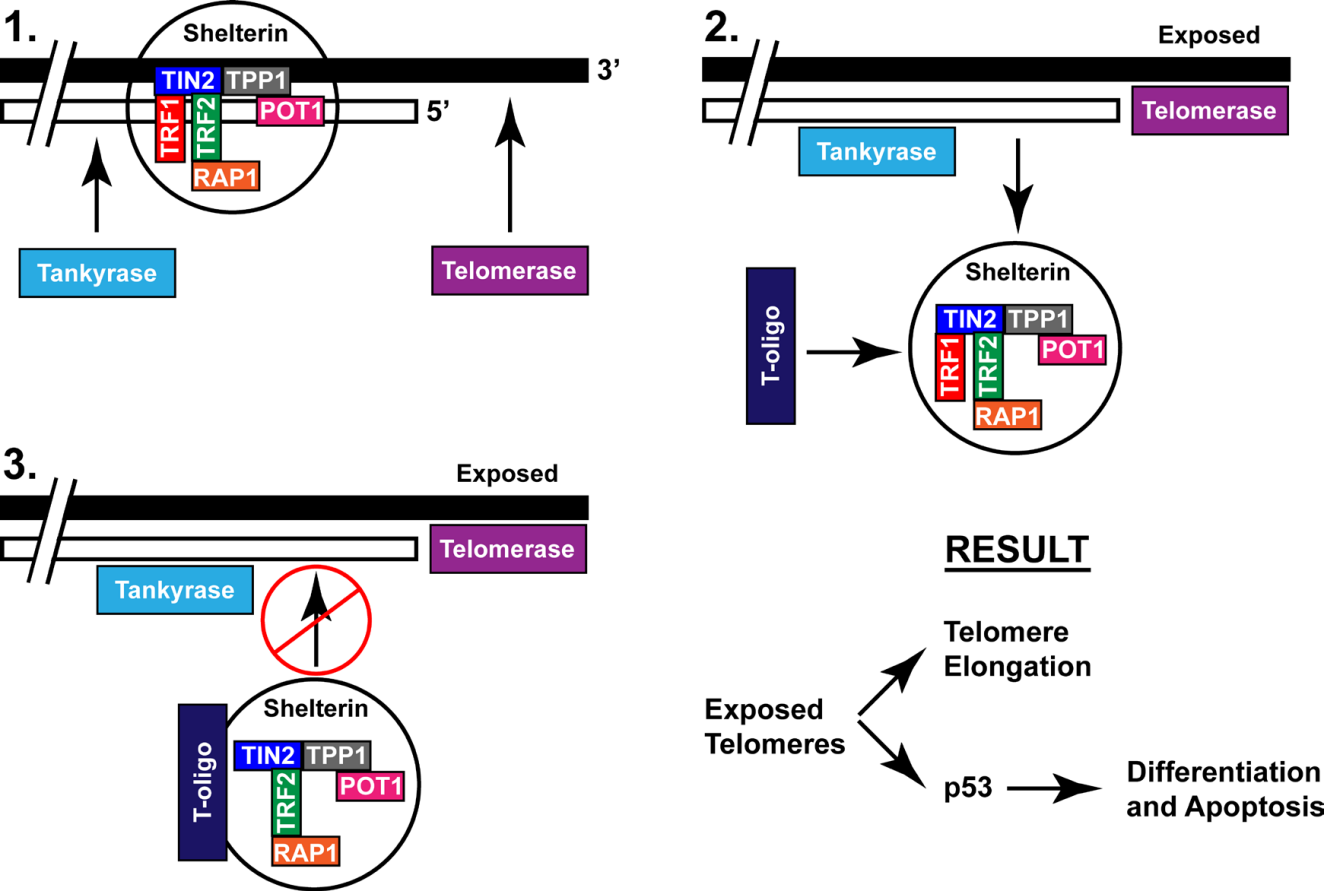
Proposed mechanism of T-oligo induced activation of the DNA damage response through recruitment of the shelterin complex The shelterin complex, composed of TRF1, TRF2, TIN1, POT1, TPP1, and RAP1 stabilizes the telomere and prevents telomere overhang exposure. Tankyrase causes dissociation of shelterin through PARsylation of TRF1, leading to telomere overhang disruption. T-oligo is then able to bind the shelterin complex and maintain exposure of the telomere overhang which leads to either telomere elongation or DNA damage responses including apoptosis or differentiation.

At present the role of tankyrase-1 in T-oligo mediated DNA damage responses is unclear. We suggest that T-oligo binds to shelterin associated proteins following PARsylation of TRF1, and recruits the shelterin complex away from the telomere (Fig 6). In this uncapped state, telomeres are exposed, thus providing an opportunity for recognition as DNA damage foci by ATM or WRN [19, 24]. Alternatively, this allows for binding of the telomere by telomerase, previously suggested by evidence that T-oligo increases mean telomere length [19], possibly through a transient increase in telomerase activity [42]. An increase in mean telomere length may also explain our finding of increased TRF2 expression after exposure to T-oligo since elongated telomeres require increased TRF2 for stability [6]. This is in contrast to treatment with TRF2DN which results in telomere degradation [4], or other studies that show upregulation of TRF2 maintains the telomere duplex structure. In the present experiment, we propose that inhibition of tankyrase-1 maintains the integrity of the telomere, thus completely blocking T-oligo induced DNA damage responses and suggesting that destabilization of the telomere overhang is a requirement for T-oligo to act as a therapeutic agent. These results suggest that T-oligo functions to prevent restabilization following dissociation of TRF1 from the shelterin complex, leading to disruption of the T-loop and downstream DNA damage responses. We therefore suggest additional experiments involving disruption of TRF1 to further define its role in T-oligo-induced DNA damage responses.

In addition to PARsylating TRF1, tankyrase-1 contributes many functions to cellular physiology including PARsylation of axin, targeting it for ubiquitination and degradation, and inhibiting the Wnt pathway causing growth inhibition [18] and regulation of mitosis [44]. At present, there is no data to suggest that T-oligo could function to modulate these other pathways; however these findings suggest new avenues of research.

In this study, we document considerable advances in understanding the mechanism of T-oligo induced DNA damage responses. p53 is shown to be an important contributor to T-oligo induced apoptosis and differentiation in p53-expressing melanoma cells. Further, inhibition of tankyrase-1 prevents T-oligo induced DNA damage responses, suggesting a new paradigm in understanding T-oligo's mechanism of action. These results are novel, important and provide new insight into telomere biology and the mechanism whereby T-oligo induces DNA damage responses.

## MATERIAL AND METHODS

### Oligonucleotides

11-base DNA oligonucleotides homologous (pGTTAGGGTTAG) (T-oligo) and complementary (pCTAACCCTAAC) (complementary oligo) to the 3' telomere overhang sequence were obtained from Midland Certified Reagent Company (Midland, TX) for cell culture experiments.

### Cell culture

MU, PM-WK, and MM-MC melanoma cells were obtained by explant culture [45] and grown in MEM (#32561-037) with 10% FBS (v/v) (#16000-044) from Gibco (Gaithersburg, MD, USA) and 1% antibiotic/antimycotic (v/v) (#15240) obtained from Invitrogen (Grand Island, NY) as described previously [3].

### Immunoblotting and antibodies

Melanoma cells were grown in MEM media containing 5% FBS and treated with diluent (water), 40 μM complementary oligonucleotide, or 40 μM T-oligo for various time points. After which, cells were harvested and protein lysates immunoblotted as described previously [46]. p53 (pantropic) antibody (clone DO-1, #OP43A) was obtained from EMD Millipore (Billerica, MA). phospho-p53 (Ser 15) (#:9284) polyclonal antibodies, cleaved Caspase-3 (Clone 5A1E, Rabbit mAb #9664), cleaved Caspase-7 (Clone D6H1, Rabbit mAb #8438), and cleaved Caspase-9 (Clone D2D4, Rabbit mAb #7237) were purchased from Cell Signaling Technology (Danvers, MA). For detection of differentiation, the following antibodies were used; tyrosinase monoclonal antibody (clone T311: Novacastra™, Leica Biosystems Newcastle upon Tyne, UK), TRP-1 (sc 10443) and TRP-2 (sc 10452) polyclonal antibodies (Santa Cruz Biotechnology, Santa Cruz, CA), and MART-1/Melan-A monoclonal antibody (clone M2-7C10: Signet, Dedham, MA). The TRF1 antibody was a generous gift from Dr. Jan Karlseder, and TRF2 antibody was purchased from (#NB110-57130: Novus Biologicals, Littleton, CO). Beta-actin antibody (#A-5441), Sheep anti-Mouse IgG (#NA931V), and Donkey anti-Rabbit IgG (#NA934V) were purchased from GE healthcare (Piscataway, NJ), and all other chemicals were obtained from Sigma Aldrich (St. Louis, MO). Densitometry was performed using ImageJ software (National Institutes of Health, Bethesda, Maryland, USA).

### Transfection of p53 siRNA

MU melanoma cells were transfected with four pooled siRNA duplexes, from Dharmacon (Lafayette, CO) against p53 (#M-003329-03-0005) in optiMEM (#11058-021, Invitrogen, Carlsbad, CA) using oligofectamine (#12252-011: Invitrogen, Carlsbad, CA). Mock transfection was done in parallel using signal silence control siRNA (#6568S: Cell Signaling Technology, Beverly, MA).

### Determination of Apoptosis

Melanoma cells were cultured as described previously and transfected with siRNA for 12 hrs and subsequently treated with diluent (water), 40 μM T-oligo, or 40 μM complementary oligo for 72 hrs. Cells were then stained with propidium iodide (#81845: Sigma-Aldrich; St. Louis, MO) and analyzed by a Becton Dickinson FACS Scan. Apoptosis was studied by determining sub G_o_/G_1_ DNA content by FACS analysis as described previously [3]. Statistical significance was determined using the paired Student's T-test with significance established at alpha=0.05.

Apoptosis was also determined by evaluation of caspase-3 activity using the CaspACE assay system (#G7351) from Promega (Madison, Wisconsin). Quadruplicate cultures of melanoma cells were treated with diluent (water), 40 μM control oligonucleotide, or 40 μM T-oligo for 72 or 96 hrs. Cells were then collected by centrifugation, washed and lysed by repeated freeze/thaw cycles. The lysate was then clarified by centrifugation and the supernatant was used for protein estimation and for the caspase assay. This assay uses Ac-DEVD-pNA as a substrate and colorimetrically measures the release of free pNA. Statistical significance was determined using the paired Student's T-test with significance established at alpha=0.05.

### Tankyrase-1 inhibition

For measuring the effect of tankyrase-1 inhibitors on T-oligo induced cellular proliferation in MU melanoma cells, triplicate cell cultures were plated at 6×10^4^ cells in 60 mm^2^ dishes in MEM with 10% FBS and treated with tankyrase-1 inhibitors, XAV939 (5μM) or 3AB (20mM), or equal amount of diluent (water), and a final concentration of 40 μM T-oligo, complementary oligo, or equal amount of diluent (water). After 24 hrs, cells were prepared for immunoblotting. After 96 hrs, cells were trypsinized and counted using a hemocytometer in the presence of trypan blue. Statistical significance was determined using the one-way ANOVA with significance established at alpha=0.05.
